# Prevalence, Characteristics, and Risk Factors of Retinal Hemorrhage among Full-Term Neonates in Southern China

**DOI:** 10.3390/ijerph192113927

**Published:** 2022-10-26

**Authors:** Tingting Yang, Rongsheng Hu, Jiansu Chen, Yamei Lu, Yonglong Guo, Yao Liu, Ruixia Yu, Guangming Jin

**Affiliations:** 1 Department of Ophthalmology, First Affiliated Hospital of Jinan University, Jinan University, Guangzhou 510632, China; 2 Department of Ophthalmology, The Sixth Affiliated Hospital of Guangzhou Medical University, Qingyuan People’s Hospital, Qingyuan 511518, China; 3 Department of Urology, The Sixth Affiliated Hospital of Guangzhou Medical University, Qingyuan People’s Hospital, Qingyuan 511518, China; 4 Institute of Ophthalmology, Medical College, Jinan University, Guangzhou 510632, China; 5 State Key Laboratory of Ophthalmology, Zhongshan Ophthalmic Center, Guangdong Provincial Key Laboratory of Ophthalmology and Visual Science, Guangdong Provincial Clinical Research Center for Ocular Diseases, Sun Yat-sen University, Guangzhou 510060, China

**Keywords:** retinal hemorrhage, prevalence, risk factors, neonates

## Abstract

Neonatal retinal hemorrhage (RH) is the most common ocular fundus disease among newborns. Early detection and timely intervention are vital for reducing the risk of visual impairment caused by RH. However, little is known about the prevalence, characteristics, and risk factors of RH in southern China. Full-term infants born in Qingyuan City during the first 10 days of each month in 2021 were included in this study. All infants underwent RetCam III retinal examinations. Detailed information on retinal hemorrhage, including involved eyes, bleeding severity, and affected area (extrafoveal macula, fovea, or optic disc), and clinical information on the neonates and their mothers was collected. The results showed that among the 1072 eligible neonates, 266 (24.8%) had neonatal retinal hemorrhage. Consistent bilateral retinal hemorrhage severity was observed in 83.2% of the cases. The prevalence of optic disc involved RH, extrafoveal macular involved RH and foveal involved RH were 23.7%, 81.2% and 2.63%, respectively. Multivariate logistic regression analysis showed that lower birth weight (OR, 0.63; 95% CI, 0.40–0.99; *p* < 0.05) and vaginal delivery (OR, 20.6; 95% CI, 9.10–46.5; *p* < 0.001) were risk factors of neonatal RH. The area under the ROC curve of vaginal delivery, combined with birth weight, as predictors of neonatal RH was 0.73, with 85.3% sensitivity and 23.9% specificity. The birth weight cutoff was 3460 g. Our results suggested that neonatal RH is common in full-term neonates in southern China. It usually has the same severity in both eyes and mostly involves the extrafoveal macular region. Vaginal delivery and low birth weight are risk factors for neonatal RH.

## 1. Introduction

Neonatal retinal hemorrhage was first described by Von Jäger (1861) [1]. Its reported prevalence ranges from 2.6% to 50%, which makes it the most common ocular fundus disease among newborns [1,2,3,4]. Although neonatal retinal hemorrhage is generally considered benign and is mostly spontaneously absorbed within one month [3,5,6], severe hemorrhage, or hemorrhage involving the fovea, can lead to prolonged hemorrhage absorption time and retinal toxicity, resulting in visual disorders, such as ametropia, strabismus, and amblyopia [7,8,9,10]. Early detection and timely follow-up and intervention strategies are vital for reducing the risk of visual impairment.

Considering the prevalence, characteristics, and risk factors of RH is important for disease management. Previous studies based on non-neonatal digital wide-field fundus imaging systems have been conducted [1,4,6,9,11,12,13,14,15,16,17,18,19,20,21,22,23,24,25,26]. Emerson investigated the prevalence and disappearance rate of retinal hemorrhage in 149 healthy neonates using indirect ophthalmoscopy and found that 34% of the subjects had retinal hemorrhage of varying severity [6]. Vinekar used RetCam to screen 1021 full-term infants and reported that the prevalence of neonatal retinal hemorrhage was only 2.4% [27]. Cho used wide-field digital retinal imaging and reported a prevalence of 23.2% among 56,247 healthy neonates [8]. Moreover, they found that neonatal retinal hemorrhage was significantly associated with natural vaginal delivery and that severe hemorrhage showed common macular and/or optic disc involvement. 

However, China, which has a large population with an annual birth rate of 12% [28], has limited data on neonatal retinal hemorrhage [29,30,31,32,33], and the few existing studies have not fully evaluated the risk factors associated with mothers and newborns. Moreover, the descriptions of the characteristics of retinal hemorrhage are not sufficiently detailed, which limits our knowledge of the condition and the development of prevention and treatment strategies. ^28^ To provide a basis for screening and prevention strategies for neonatal retinal hemorrhage, we conducted a comprehensive investigation of retinal hemorrhage in full-term neonates undergoing RetCam III retinal examinations and evaluated its prevalence, clinical characteristics, and possible influencing factors.

## 2. Materials and Methods 

### 2.1. Design and Subjects

**Figure 1:**
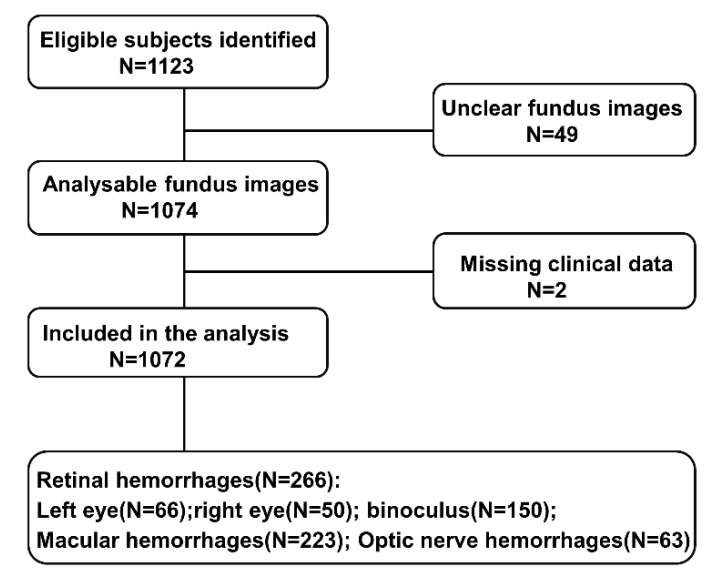
Flowchart of participants included and excluded from analysis for retinal hemorrhage.

This study involved neonates born in Qingyuan City, Guangdong Province, undergoing RetCam screening between January 2021 and December 2021. Stratified cluster sampling was used to select subjects born during the first 10 days of each month from 3529 eligible newborns. Neonates with a gestational age below 37 weeks, bilateral absence of eyeballs, or missing clinical data were excluded. According to these criteria, 1123 subjects were initially included. After excluding 49 neonates with unclear fundus images and two with missing clinical data, a final sample of 1072 was included in the analysis. A flowchart of the inclusion process is shown in Figure 1.

This study was approved by the Ethics Committee of the Sixth Affiliated Hospital of Guangzhou Medical University. All subjects’ legal guardians signed written informed consent forms before the examinations.

### 2.2. Examination Method

The newborns underwent fundus examination 24 h to one week after birth. The fundus image collection and analysis were performed by the same medical team, led by an experienced pediatric retinal surgeon and an experienced nurse. The newborns’ guardians were instructed not to provide the newborns with water or milk two hours before the examination. The examination procedure was as follows: (1) One hour before the examination, compound tropicamide eye drops were administered three times at 10-min intervals (one to two drops each time) to fully dilate the pupil. (2) Topical anesthesia was induced using proparacaine hydrochloride eye drops. (3) An eyelid opener for infants was applied. (4) The surface of the cornea was coated with ofloxacin eye gel for lubrication and protection. (5) Images of the entire retina were captured by an experienced physician using a 130° RetCam III system (Clarity Medical Systems, Inc., Pleasanton, CA, USA). The retinal images were divided into five segments: center of the optic disc, above the optic disc, below the optic disc, nasal, and temporal. (6) Topical antibiotic eye drops were administered at the end of the examination.

### 2.3. Determination and Classification of Neonatal Retinal Hemorrhage

All retinal images were reviewed independently by two experienced retinal surgeons, and a third senior retinal surgeon was consulted in cases of disagreement in interpretation. The analysis of neonatal retinal hemorrhage included the following information: involved eye, hemorrhage severity, and bleeding area (involvement of the extrafoveal macular region, fovea, or optic disc).

Hemorrhage was classified as mild, moderate, and severe according to a previously published classification [3]. According to this classification, mild cases are characterized by minimal bleeding, 1–10 bleeding spots, and a bleeding area smaller than one optic disc area; moderate cases are characterized by greater bleeding and 11–30 bleeding spots; and severe cases are characterized by massive bleeding and 31 or more bleeding spots. If the severity of retinal hemorrhage was not consistent between the two eyes, the classification was based on the eye with more severe hemorrhage.

Foveal hemorrhage was defined as any hemorrhage in the fovea (approximately the size of an optic disc within the reflection halo of the boundary membrane in the macular region in digital photographs). Extrafoveal macular hemorrhage was defined as bleeding in the area between the upper and lower vascular arches of the posterior pole of the retina (except in the fovea). Optic disc hemorrhage was defined as any hemorrhage involving the optic disc (a round orange-red disc about 3 mm from the nasal side of the macula).

### 2.4. Data Collection

Information on each newborn and mother was collected. Data on the neonates included gender, gestational age (in weeks), birth weight (in grams), Apgar score, screening age (in hours), birth position, umbilical cord around the neck (yes/no), fetal distress (yes/no), and scalp hematoma (yes/no). Data on the mothers included prepartum body mass index, gestational hypertension (yes/no), gestational diabetes mellitus (yes/no), mode of delivery (vaginal or cesarean section), number of births, amniotic fluid index, instrument midwifery (yes/no), perineum tear (yes/no), premature membrane rupture (yes/no), and placental abruption (yes/no).

### 2.5. Statistical Analysis

All data were analyzed using Stata 15 software (StataCorp, College Station, TX, USA). Analysis of variance was used for comparisons of normally distributed quantitative data between the three hemorrhage severity groups, and the rank sum test was used for comparisons of qualitative data and non-normally distributed quantitative data. The chi-squared test was used to compare categorical variables between the three groups. Binary logistic regression was used to evaluate factors associated with neonatal retinal hemorrhage. A *p*-Value of <0.05 was considered statistically significant. A receiver operating characteristic (ROC) curve was used to evaluate the value of the related factors.

## 3. Results 

Of the 1072 included neonates, 266 (24.8%) had neonatal retinal hemorrhage. Table 1 shows the demographic characteristics of the neonates in the three retinal hemorrhage severity groups. The mean birth weight of the severe retinal hemorrhage group (3177.3 ± 341.8 g) was significantly higher than that of the mild (3139.4 ± 366.6 g; *p* < 0.05) and moderate (3143.5 ± 370.9 g; *p* < 0.05) severity groups. In all three groups, the proportion of first-born babies was higher than that of non-first-born babies (mild hemorrhage group: 54.4% vs. 45.6%; moderate hemorrhage group: 67.5% vs. 32.5%; severe hemorrhage group: 69.9% vs. 30.1%; all *p* < 0.05).

Table 2 shows an analysis of the consistency of retinal hemorrhage severity between the two eyes. Consistent bilateral retinal hemorrhage severity was observed in 83.2% of the patients. The moderate severity group had the lowest consistency rate.

**Figure 2:**
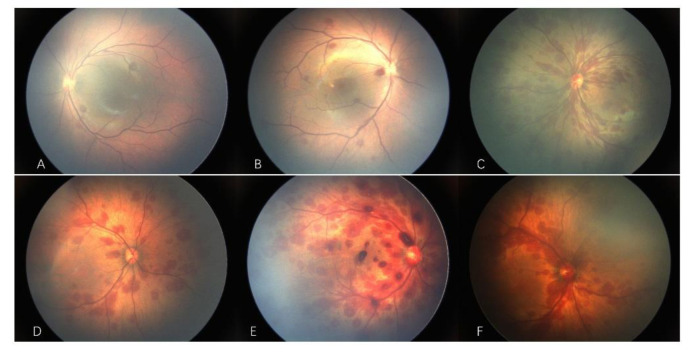
Representative fundus photographs of newborns with retinal hemorrhage. (**A**) Mild, (**B**) moderate, and (**C**) severe hemorrhage. Hemorrhage with (**D**) extrafoveal macular, (**E**) foveal, and (**F**) optic disc involvement.

Figure 2 shows images of neonatal retinal hemorrhage of varying severity and hemorrhage with extrafoveal macular, foveal, and optic disc involvement. Table 3 shows the characteristics of neonatal retinal hemorrhage by severity. There were significant differences in extrafoveal macular area and optic disc involvement between the three hemorrhage severity groups (all *p* < 0.001). In all three groups, the rate of extrafoveal macular area involvement in severe hemorrhage group was higher than in the other two groups. In the severe hemorrhage group, the involvement rate was almost 100%. The rate of optic disc involvement was lower in all groups, but it was higher in the severe hemorrhage group than in the other two groups. Only seven patients, four of whom were in the severe hemorrhage group, exhibited foveal involvement.

Table 4 presents the results of the logistic regression analyses of factors related to neonatal retinal hemorrhage. Multivariate logistic regression analysis showed that low birth weight (OR, 0.63; 95% CI, 0.40–0.99; *p* < 0.05) and vaginal delivery (OR, 20.6; 95% CI, 9.10–46.5; *p* < 0.001) were significantly associated with neonatal retinal hemorrhage.

**Figure 3:**
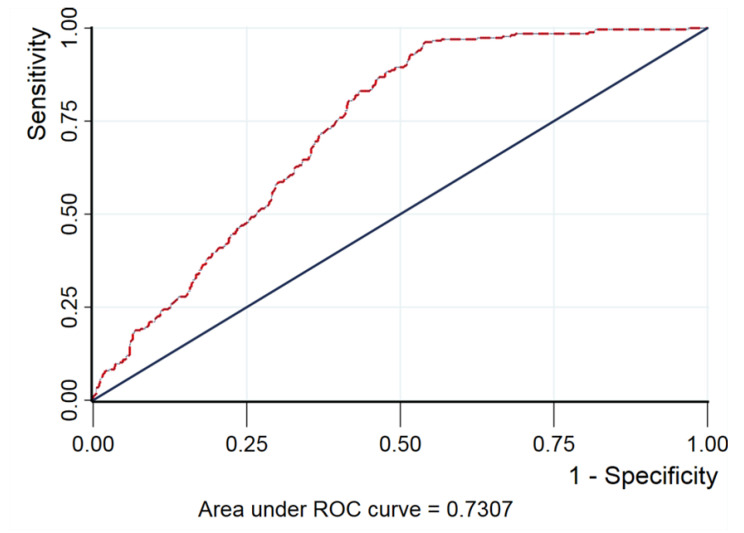
Predictive value of vaginal delivery and birth weight for neonatal retinal hemorrhage shown by ROC curve (AUC = 0.7307).

Figure 3 shows the ROC curve of the factors related to retinal hemorrhage. The area under the ROC curve of vaginal delivery, combined with birth weight, as a predictive factor of neonatal retinal hemorrhage was 0.73, with 85.3% sensitivity and 23.9% specificity. The birth weight cutoff was 3460 g. 

## 4. Discussion

In this study, about a quarter of full-term newborns had retinal hemorrhage. Consistent bilateral retinal hemorrhage severity was observed in 83.2% of the patients. Most subjects exhibited extrafoveal macular involvement, and nearly a quarter showed optic disc involvement, whereas foveal involvement was less common. Vaginal delivery and a birth weight of less than 3460 g showed good predictive value for neonatal retinal hemorrhage.

The prevalence of neonatal retinal hemorrhage in this study was 24.8%. This is comparable to the rates reported by some previous studies (20.2–24.5%) [8,22,30,32,34], but significantly higher than those reported by other studies (2.4–14.5%) [9,26,27,35,36,37], and lower than those reported by others (34–39.4%) [4,6,20,25,31]. Despite attempts to explain the reasons for these significant differences, no statistically proven explanation has been provided. Giles et al. [1] observed retinal hemorrhage in 40% of neonates examined one hour after birth. The rate dropped to 36% when the examination was performed 24 h later, 25% when it was performed 48 h later, and 20% when it was performed 72 h later. Another study reported a prevalence of 18.9% within the first 24 h of birth, 12.5% after two to three days, and only 2.6% after three to five days [13]. Thus, the later the time of examination, the lower the prevalence of neonatal retinal hemorrhage. Moreover, Callaway found that self-identified Hispanic or Latino ethnicity was protective against neonatal retinal hemorrhage [34]. Therefore, the possible reasons for the considerable differences in the prevalence of neonatal retinal hemorrhage include examination time, ethnic differences and screening technology and methods [1,2,20,25,27,34,38].

We found that the severity of neonatal retinal hemorrhage was generally consistent (83.2%) between the two eyes. This is comparable to the 71.4% consistency reported by Cho et al. [8]. Conversely, Emerson et al. [6] found no consistency. In our study, 95.9% (255/266) of the neonates with retinal hemorrhage were delivered vaginally. Retinal hemorrhage mainly occurs as the head passes through the birth canal from the lower segment of the uterus, during which process both eyes are pressured by the birth canal with roughly equal probability and intensity. This may explain the consistent hemorrhage severity in both eyes observed in our study.

In our study, 81.2% of the neonates with retinal hemorrhage exhibited extrafoveal macular involvement. This is consistent with Callaway et al., who reported that neonatal retinal hemorrhage involved the macula in 82.9% of the patients [34]. We also found that in the severe hemorrhage group, almost all patients exhibited extrafoveal macular involvement. Cho et al. found that in neonates with severe retinal hemorrhage, the proportion of simultaneous macula and optic disc involvement was 96.68% [8]. The reason neonatal retinal hemorrhage mostly involves the macular area may be that the retinal blood vessels in this area are more sensitive to ischemia and hypoxia.

Conversely, we found a low rate of foveal involvement (2.63%). This is consistent with the rates reported by Zhao et al., (2.72%) [30], Goyal et al., (2.61%) [36], Ma et al., (3.13%) [37], and Fei et al., (4.55%) [38], but significantly lower than those reported by Callaway et al., (14.6%) [34], Li et al., (17.18%) [39], and Simkin et al., (16%) [35]. Even mild hemorrhage involving the fovea may be accompanied by degenerative changes or exudative or glial scarring [7]. Schenker et al. reported that up to 11% of children exhibiting retinal hemorrhage at birth showed changes in the granular macula by the age of 3 years [40]. Therefore, although neonatal retinal hemorrhage involves the fovea less frequently, it requires special attention, as it may lead to visual impairment, such as congenital strabismus, amblyopia, and nystagmus.

In this study, 23.7% of the patients with neonatal retinal hemorrhage exhibited optic disc involvement. This rate is significantly lower than the rate of 68.3% reported by Callaway et al. [34]. Such differences may be due to differences in ethnicity, screening times, sample sizes, and delivery styles.

In this study, vaginal delivery was a risk factor for neonatal retinal hemorrhage. Previous studies have also reported that vaginal delivery is significantly associated with neonatal retinal hemorrhage [1,4,6,8,12,26,29,30,31,34]. Callaway et al. found that 29.6% of babies delivered vaginally exhibited retinal hemorrhage, compared to 5.2% of babies delivered by cesarean section [34]. Cho et al. reported rates of 94.34% and 5.66%, respectively [8]. Zhao et al. found that natural vaginal delivery positively correlated with the occurrence of neonatal retinal hemorrhage, whereas cesarean delivery showed a negative correlation [30]. During vaginal delivery, fetal head compression leads to a sharp increase in intracranial pressure and peripheral central retinal vein congestion, expansion, or even rupture, leading to retinal hemorrhage [9,41]. Moreover, infants have more branch points and a smaller segmental length of immature microvessels, experience greater stress and tension during the traumatic load period, and are more prone to retinal hemorrhage than adults [42].

In our study, birth weight was also significantly associated with neonatal retinal hemorrhage. Yanli et al., and Pu et al., also reported a significant correlation between birth weight and neonatal retinal hemorrhage [31,33]. Conversely, Emerson et al., Giles et al., Svenningsen et al., Zhao et al., Gonzalez et al., Bergen et al., Callaway et al., and Cho et al., found no significant correlation [1,6,8,16,19,25,30,34]. Newborns with a low birth weight may be more likely to develop retinal hemorrhage due to their less mature retinal blood vessels and greater vascular vulnerability. However, a low birth weight may also be related to the choice of delivery method. The higher prevalence of retinal hemorrhage among neonates with a lower birth weight may be because more of these babies are delivered vaginally. Hsu et al. reported that among babies delivered by cesarean section, the proportion of babies with a birth weight of more than 3500 g was higher than that of babies with a low birth weight [43].

It has been reported that neonatal retinal hemorrhage can be prevented using vitamins B, K, and E and other “nontoxic antifragility substances” [13]. In the previous study, the prevalence of neonatal retinal hemorrhage (34%) in an untreated group of pregnant mothers was significantly higher than in a group receiving an “antifragility substance” (7%) [13].

Overall, our findings suggest that vaginal delivery, combined with low birth weight, can be used to predict neonatal retinal hemorrhage. Screening efficiency can be improved by paying attention to these two factors, especially in economically underdeveloped areas.

This study has certain limitations. Due to its retrospective design, the neonates with retinal hemorrhage were not followed up. Moreover, all patients were from the Qingyuan region, as representative of southern China. Multicenter studies with larger samples are needed to generalize the results. One of the strengths of this study is that we comprehensively evaluated factors affecting neonatal retinal hemorrhage in both the infants and their mothers. Moreover, we characterized retinal hemorrhage in detail. Finally, we reported, for the first time, that vaginally delivered newborns, combined with a birth weight of less than 3460 g, were at greater risk of retinal hemorrhage.

## 5. Conclusions

Retinal hemorrhage is one of the most common ocular abnormalities among neonates in southern China. Neonatal retinal hemorrhage is characterized by consistent severity in both eyes and low foveal involvement rates. Vaginal delivery and low birth weight (less than 3460 g) are risk factors for retinal hemorrhage among full-term neonates. Ophthalmologists should increase screening and fundus examinations of high-risk newborns to ensure early detection of severe retinal hemorrhage and prevent possible visual impairment.

## Figures and Tables

**Table 1:** Demographic characteristics of the subjects according to retinal hemorrhage severity.

Characteristics	Total (*n* = 1072)	Mild RHs (*n* = 103)	Moderate RHs (*n* = 40)	Severe RHs (*n* = 123)	*p*-Value
**Fetal information**					
**Gender**					
**Male (*n*, %)**	608(56.7)	49(47.6)	20(50)	71(57.7)	0.294
**Female (*n*, %)**	464(43.3)	54(52.4)	20(50)	52(42.3)	
**Gestational age (weeks)**	39.3 ± 0.99	39.3 ± 0.96	39.1 ± 1.01	39.3 ± 0.98	0.770
**Birth weight (g)**	3209.5 ± 363.4	3139.4 ± 366.6	3143.5 ± 370.9	3177.3 ± 341.8	0.047
**Agar score (IQR)**	10(10, 10)	10(10, 10)	10(10, 10)	10(10, 10)	0.999
**Age at examination (days)**					
**<48 h**	639(59.6)	84(81.6)	30(75.0)	103(83.7)	0.464
**>48+ h**	433(40.4)	19(18.4)	10(25.0)	20(16.3)	
**Position of the fetus**					
**Left occipitoanterior**	726(67.7)	74(71.8)	28(70.0)	94(76.4)	0.626
**Other**	346(32.3)	29(28.2)	12(30.0)	29(23.6)	
**UCAN**					
**No**	738(68.8)	75(72.8)	30(75.0)	88(71.5)	0.911
**Yes**	334(31.2)	28(27.2)	10(25.0)	35(28.5)	
**Fetal distress**					
**No**	1004(93.7)	98(95.2)	37(92.5)	120(97.6)	0.338
**Yes**	68(6.34)	5(4.85)	3(7.50)	3(2.44)	
**Scalp hematoma**					
**No**	1032(96.3)	97(94.2)	35(87.5)	118(95.9)	0.149
**Yes**	40(3.73)	6(5.83)	5(12.5)	5(4.07)	
**Mother’s information**					
**BMI before production**	26.3 ± 3.23	26.0 ± 3.06	26.6 ± 3.71	25.9 ± 3.11	0.230
**PIH**					
**No**	1042(97.2)	103(100)	40(100)	122(97.2)	0.558
**Yes**	30(2.80)	0	0	1(0.81)	
**GDM**					
**No**	918(85.6)	92(89.3)	30(75.0)	108(87.8)	0.067
**Yes**	154(14.4)	11(10.7)	10(25.0)	15(12.2)	
**Delivery method, *n* (%)**					
**Vaginal**	689(64.3)	98(95.2)	38(95.0)	119(96.8)	0.798
**Cesarean section**	383(35.7)	5(4.85)	2(5.00)	4(3.25)	
**Parity**					
**First birth**	358(33.4)	47(45.6)	13(32.5)	37(30.1)	0.046
**Non first birth**	714(66.6)	56(54.4)	27(67.5)	86(69.9)	
**Amniotic fluid index**					
**Normal**	953(88.9)	92(89.3)	38(95.0)	108(87.8)	0.435
**Abnormal**	119(11.1)	11(10.7)	2(5.00)	15(12.2)	
**Use obstetric apparatus**					
**No**	1059(98.8)	101(98.1)	38(95.0)	123(100)	0.070
**Yes**	13(1.21)	2(1.94)	2(5.00)	0	
**Laceration of perineum**					
**No**	457(42.6)	19(18.5)	8(20.0)	14(11.4)	0.234
**Yes**	615(57.4)	84(81.6)	32(80.0)	109(88.6)	
**PROM**					
**No**	820(76.5)	76(73.8)	30(75.0)	101(82.1)	0.291
**Yes**	252(23.5)	27(26.2)	10(25.0)	22(17.9)	
**Placental abruption**					
**No**	1069(99.7)	102(99.0)	40(100)	123(100)	0.452
**Yes**	3(0.28)	1(0.97)	0	0	

**Abbreviations:RHs** = retinal hemorrhages; **IQR** = interquartile range; **UCAN** = umbilical cord around the neck; **PIH** = pregnancy-induced hypertension syndrome; **GDM** = gestational diabetes mellitus; **PROM** = premature rupture of membrane. Analysis of variance and the rank sum test was used for comparisons of the three hemorrhage severity groups. *p*-Value: statistically significant at <0.05.

**Table 2:** Bilateral consistency rates of neonatal retinal hemorrhage severity.

Left Eye	Right Eye				
	None	Mild RHs	Moderate RHs	Severe RHs	Total
**None**	806(92.4)	38(46.3)	4(13.8)	8(8.99)	856(79.9)
**Mild RHs**	46(5.28)	20(24.4)	7(24.1)	18(20.2)	91(8.49)
**Moderate RHs**	13(1.49)	5(6.10)	10(34.5)	7(7.87)	35(3.26)
**Severe RHs**	7(0.80)	19(23.2)	8(27.6)	56(62.9)	90(8.40)
**Total**	872(100)	82(100)	29(100)	89(100)	1072(100)

**Abbreviations: RHs:** Retinal hemorrhages.

**Table 3:** Characteristics of eyes with retinal hemorrhage according to severity.

	Total	Mild RHs	Moderate RHs	Severe RHs	*p*-Value
**Eyes with R** **Hs**	266	103 (9.61%)	40(3.73%)	123(11.5%)	
**All Macular Hemorrhages**	223	57(25.6)	37(17.5)	127(56.9)	
**Extrafoveal involvement**					
**Yes**	216(81.2)	56(54.4)	37(92.5)	123(100)	<0.001
**No**	50(18.8)	47(45.6)	3(7.5)	0	
**Foveal involvement**					
**Yes**	7(2.63)	1(0.97)	2(5.00)	4(3.25)	0.338
**No**	259(96.8)	102(99.0)	38(95.0)	119(96.8)	
**Optic involvement**					
**Yes**	63(23.7)	2(1.94)	3(7.50)	58(47.2)	<0.001
**No**	203(76.3)	101(98.1)	37(92.5)	65(52.8)	

**Abbreviations: RHs:** Retinal hemorrhages; The rank sum test was used for comparisons of the three hemorrhage severity groups. *p*-Value: statistically significant at <0.05.

**Table 4:** Univariate and multivariate logistic regression analyses of factors related to neonatal retinal hemorrhage.

Factors	Univariate Analysis		Multivariate Analysis	
	OR (95%CI)	*p*	OR (95%CI)	*p*
Fetal information				
Female	1.27(0.96, 1.68)	0.091		
Gestational age (days)	1.00(0.87, 1.15)	0.987		
Birth weight (kg)	0.58(0.39, 0.85)	0.006	0.63(0.40, 0.99)	0.043
Agar score	0.88(0.59, 1.33)	0.558		
Age at examination				
<48 h	1.0 (reference)		1.0 (reference)	
48+ h	0.25(0.18, 0.35)	<0.001	0.97(0.64, 1.47)	0.891
Position of the fetus				
Left occipitoanterior	1.0 (reference)		1.0 (reference)	
Other	0.70(0.52, 0.96)	0.025	1.16(0.82, 1.63)	0.414
UCAN	0.77(0.57, 1.05)	0.097		
Fetal distress	0.57(0.29, 1.10)	0.092		
Scalp hematoma	3.04(0.19, 48.7)	0.433		
Mother’s information				
BMI before production	0.96(0.92, 1.00)	0.079		
PIH	0.10(0.01, 0.75)	0.025	0.21(0.03, 1.70)	0.144
GDM	0.91(0.61, 1.36)	0.656		
Delivery method				
Cesarean section	1.0 (reference)		1.0 (reference)	
Vaginal	19.9(10.7, 36.9)	<0.001	20.6(9.10, 46.5)	<0.001
First birth	1.23(0.92, 1.64)	0.170		
Amniotic fluid index	0.97(0.62, 1.52)	0.905		
Use obstetric apparatus	1.35(0.41, 4.43)	0.618		
Laceration of perineum	5.85(4.08, 8.39)	<0.001	0.92(0.56, 1.50)	0.664
PROM	0.91(0.65, 1.26)	0.556		

**Abbreviations: CI:** confidence interval; **OR:** odds ratio; **UCAN:** umbilical cord around the neck; **PIH:** pregnancy-induced hypertension syndrome; **GDM:** gestational diabetes mellitus; **PROM:** premature rupture of membrane. Binary logistic regression was used to evaluate factors associated with neonatal retinal hemorrhage.

## Data Availability

The data presented in this study are available on request from the corresponding author. The data are not publicly available due to privacy or ethical.
